# Differentiation of Pediatric Osteochondroma From Trevor′s Disease and Successful Surgical Management: A Case Report

**DOI:** 10.1155/cro/6640185

**Published:** 2026-04-18

**Authors:** Andrea Montalbano, Angelina Furyes, Matthew Spangler, Adrian Lewis

**Affiliations:** ^1^ Department of Orthopaedic Surgery, The University of Toledo, Toledo, Ohio, USA, utoledo.edu; ^2^ College of Medicine and Life Sciences, The University of Toledo, Toledo, Ohio, USA, utoledo.edu; ^3^ Promedica Russell J. Ebeid Children′s Hospital, Toledo, Ohio, USA

**Keywords:** bone tumor differentiation, case report, pediatric osteochondroma, surgical management, Trevor′s disease

## Abstract

Osteochondroma is the most common benign bone tumor; however, its presentation in the small bones of the appendicular skeleton, particularly the talus and subtalar joint, is quite rare. Although most cases of osteochondroma are asymptomatic, symptomatic presentations can lead to joint deformity, pain, swelling, tarsal tunnel syndrome, gait alterations, and a limited range of motion. Surgical resection has proven to be an effective treatment for symptomatic osteochondromas. This case report describes the management of a rare presentation of an isolated lateral osteochondroma of the talus successfully treated with surgical excision without evidence of recurrence 1.5 years postoperatively.

## 1. Introduction

Osteochondromas are the most common benign bone tumors, accounting for 20%–50% of all benign bone lesions. Osteochondromas are cartilage‐capped bony outgrowths that originate from the metaphyseal plate, predominantly affecting the distal long bones [1, 2]. Osteochondromas predominantly occur in males and individuals under 20 years of age [3]. They typically arise as isolated developmental defects but can also manifest as multiple lesions in the rare autosomal dominant condition known as multiple hereditary exostosis (MHE) [1, 2]. Typically extending from the metaphysis of long bones, common locations include the proximal tibia, proximal humerus, and distal femur [2, 3]. Notably, talar osteochondromas are rare, with fewer than 10 documented cases in the past 5 years [1, 4–9]. Osteochondromas are often asymptomatic, allowing for conservative management. However, symptomatic cases can present with joint deformity, pain, swelling, tarsal tunnel syndrome, gait alterations, and limited range of motion, often necessitating surgical intervention [3, 4, 6, 8, 9]. Reported sizes of talar osteochondromas range from 10 to 6 cm, and potential complications include neurovascular compromise, fractures, bursa formation, and malignant transformation [4, 6, 10]. Here, we present a case of a 7‐year‐old patient with an isolated lateral osteochondroma of the talus, causing gait changes, altered range of motion, and mild foot deformity. He was successfully treated with careful surgical resection of the exostosis without evidence of recurrence at 1.5 years postoperatively. Although several reports of osteochondroma may exist, a majority of cases present in adolescent or adult patients and rarely occur in the talus. Additionally, previous cases have provided limited radiologic detail in support of diagnostic confidence. Our case emphasizes the diagnostic differentiation of metaphyseal osteochondroma from epiphyseal dysplasia in a pediatric patient highlighting the use of Three‐dimensional computed tomography (3D‐CT) in conjunction with additional imaging to accurately differentiate osteochondroma from epiphyseal dysplasia, and underscores the importance of early surgical intervention to restore joint motion and preserve long‐term functionality as evidenced by excellent outcomes at 1.5 years postoperatively. Additionally, to our knowledge, this is the first case of osteochondroma to be reported in a prepubescent patient with long‐term follow‐up. This report serves to highlight management considerations of isolated talar osteochondromas and the differentiation from other musculoskeletal conditions in pediatric patients.

## 2. Case Presentation

**Figure 1:**
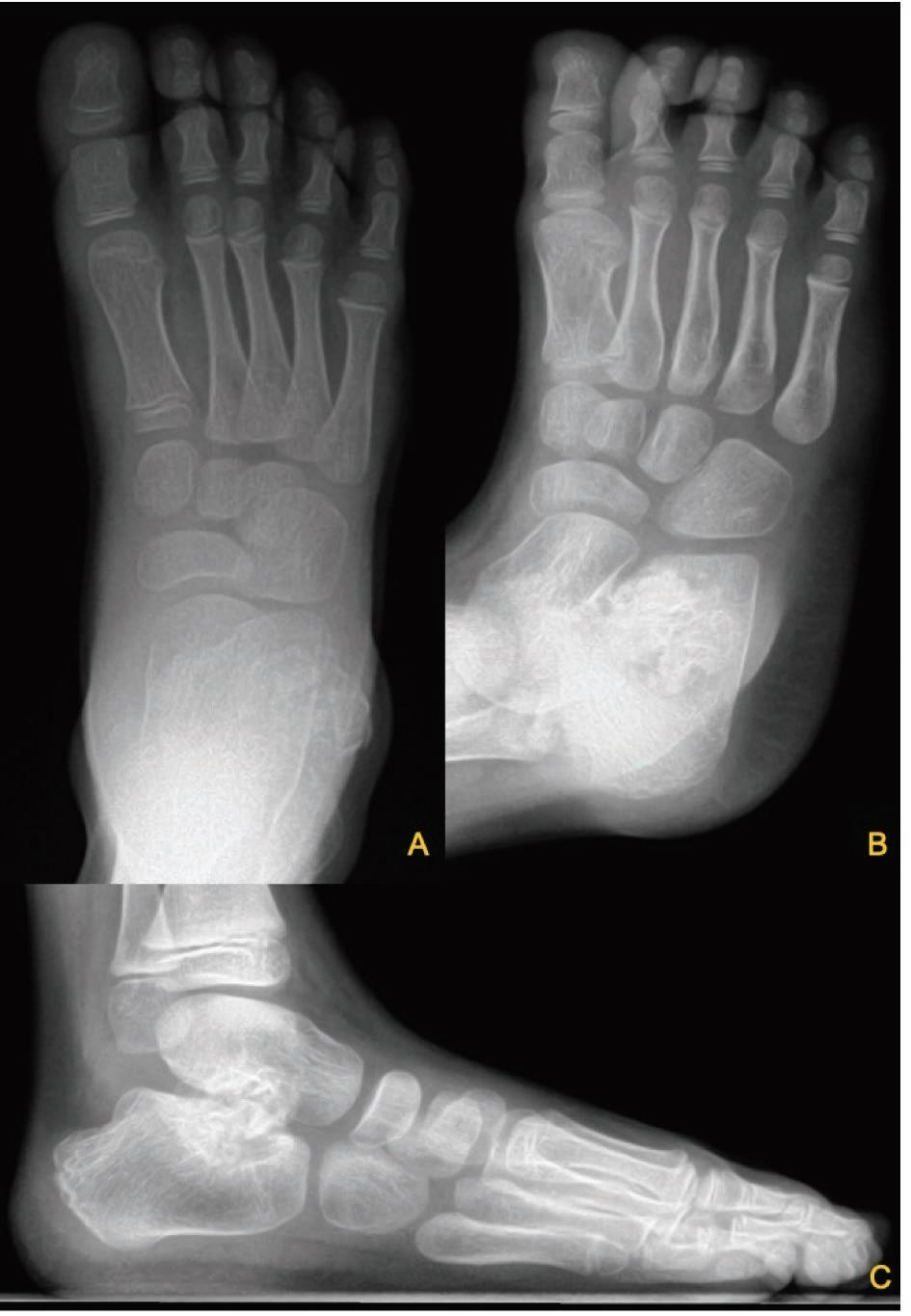
Radiographic images of the right foot including anteroposterior (A), oblique (B), and lateral (C) views demonstrating a pedunculated osseous lesion arising from the lateral talus.

**Figure 2:**
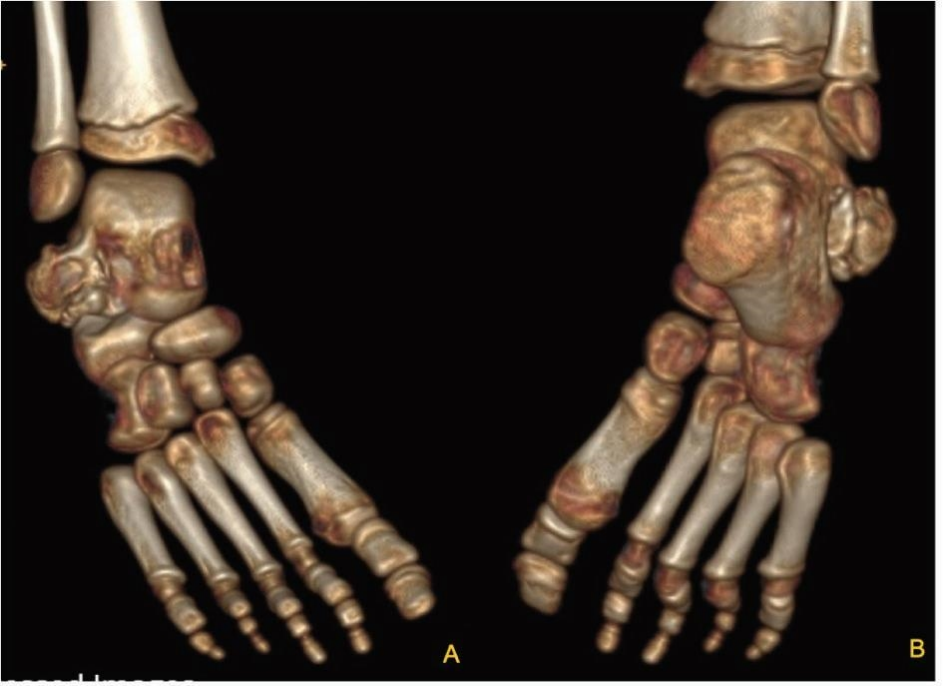
(A,B) Three‐dimensional computed tomography (3D CT) reconstructions of the right foot illustrating a pedunculated osteochondroma arising from the lateral talar body, separate from the articular surface, with extension toward the subtalar joint.

**Figure 3:**
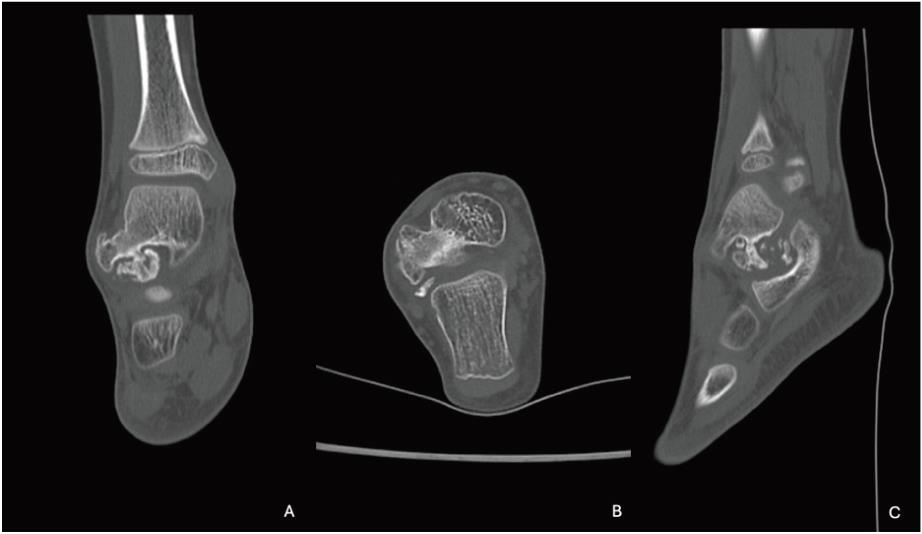
Coronal (A), axial (B), and sagittal (C) CT images demonstrating cortical and medullary continuity between the lesion and the lateral talar body, with separation from the articular surface, supporting metaphyseal origin.

**Figure 4:**
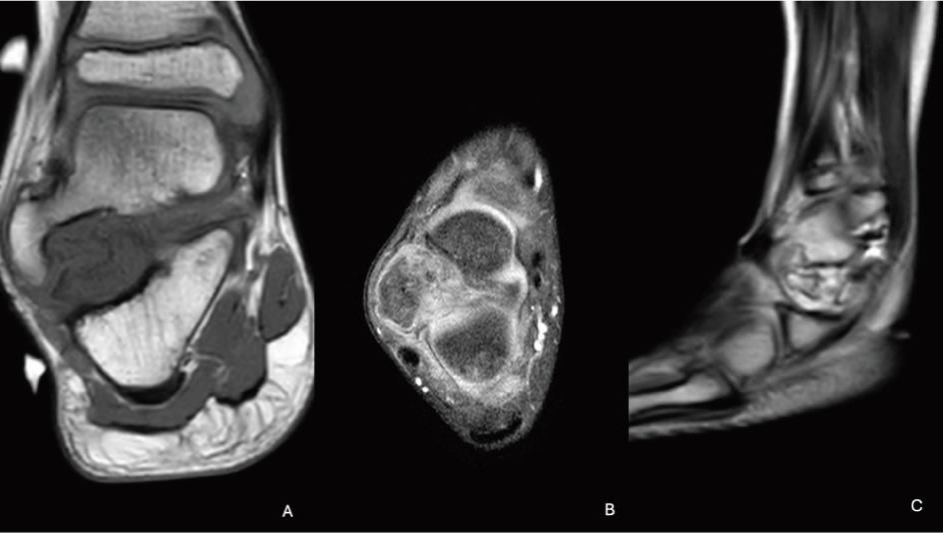
Coronal T1‐weighted (A), axial T1‐weighted (B), and sagittal T2‐weighted (C) MRI images demonstrating a cartilaginous outgrowth arising from the lateral talar body without epiphyseal communication.

We present the case of a 7‐year‐old male with no significant past medical history, referred for evaluation due to an awkward gait and a palpable bony prominence on the right foot. On physical examination, a nontender, firm mass was noted along the right lateral aspect of the talus, and the lateral border of the foot exhibited mild concavity compared with the contralateral foot. Dorsiflexion was limited to 5°, and the patient exhibited significantly reduced eversion of the joint. Inversion of the ankle, however, remained comparable with the contralateral extremity. Subtalar motion was notably decreased, and increased stiffness was observed during ambulation through the rocker phase of gait. Sensation remained intact, and no additional masses were detected. Radiographs of the right foot demonstrated a bony prominence along the lateral aspect of the talus with cortical and medullary continuity (Figure 1). A CT scan revealed a 2.0 × 1.1 × 2.7 cm lobulated, pedunculated outgrowth arising from the metaphyseal plate of the lateral talus, with infiltration into the subtalar joint. 3D‐CT reconstructions further delineated the extent of the osteochondroma, confirming its pedunculated nature and extension into the subtalar joint (Figure 2). Additional axial, coronal, and sagittal CT views demonstrated cortical and medullary continuity between the talar metaphysis and pedunculated osseous mass, confirming the diagnosis of osteochondroma (Figure 3). In addition to CT, MRI confirmed the presence of a well‐defined capped outgrowth without epiphyseal extension, further excluding the diagnosis of Trevor′s disease (Figure 4). The absence of articular surface involvement and the presence of cortical and medullary continuity with the talar body support classification as a metaphyseal‐type lesion rather than an epiphyseal‐based pathology. This multimodal radiologic approach enhanced our diagnostic confidence. Additionally, visual representation of the lesion′s morphology and relationship to surrounding structures aided significantly in preoperative planning. After discussing treatment options with the family, surgical excision was planned.

**Figure 5:**
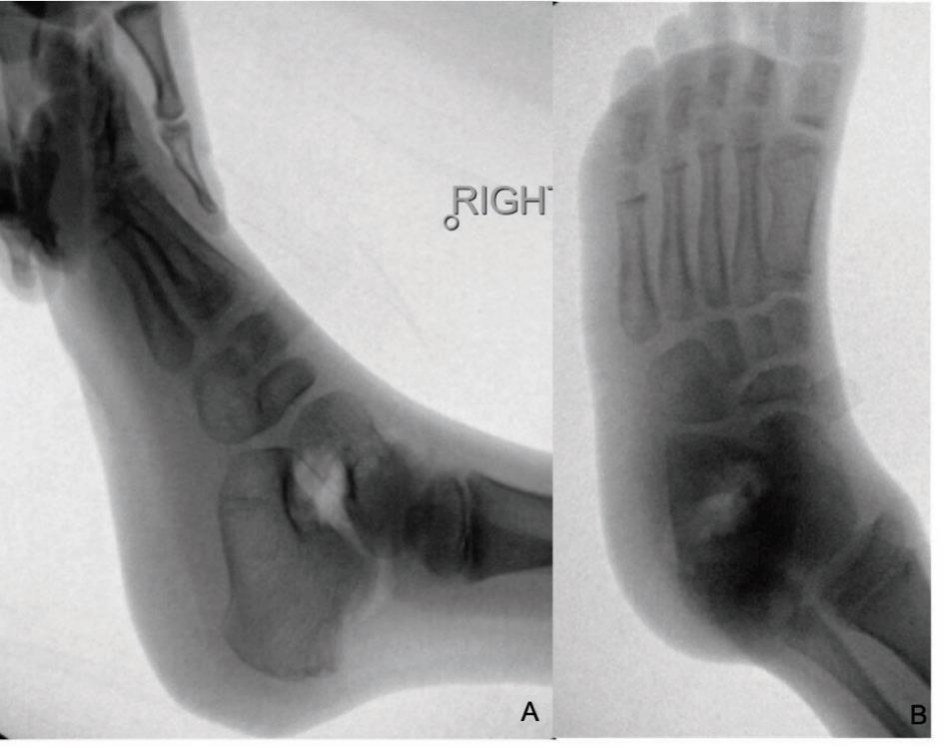
Intraoperative fluoroscopic imaging of the right foot including lateral (A) and anteroposterior (B) views confirming complete excision of the lesion.

**Figure 6:**
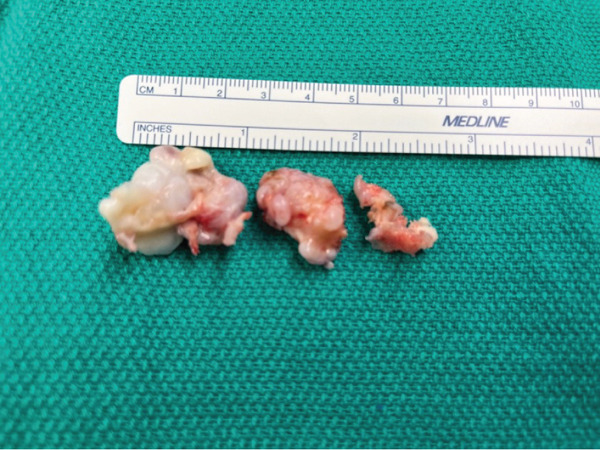
Intraoperative image of the excised osteochondroma following resection from the lateral talus and subtalar joint.

**Figure 7:**
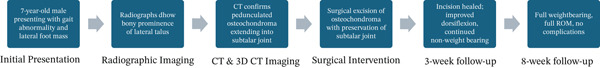
Timeline depicting clinical course from first point of care. ∗ROM: range of motion. Assessed clinically using goniometric measurement.

Surgical excision was performed through a standard lateral approach to the hindfoot. Careful dissection was utilized to identify and protect the peroneal tendons and surrounding neurovascular structures. The osteochondroma was identified as a relatively loose body in the sinus tarsi and subsequently removed without extensive debridement. The margins of the lesion were clearly delineated such that the mass essentially fell out of the wound. The mass was excised flush with the native talar cortex to minimize the risk of recurrence, and the anterior and posterior facets were visually intact following removal. No further debridement or excision was performed to prevent destabilization of the native joint. Intraoperative fluoroscopy confirmed complete excision of the osteochondroma with preservation of the subtalar joint (Figure 5). Gross examination of the excised specimen can be seen in Figure 6. An organized timeline of events is demonstrated in Figure 7.

## 3. Results

**Figure 8:**
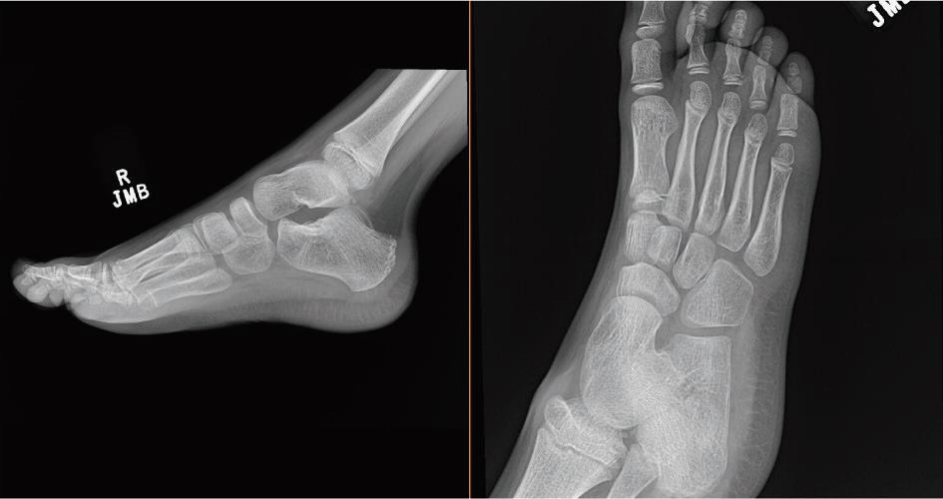
Lateral and oblique radiographs demonstrating lack of lesion recurrence and preservation of joint spaces at 1.5 years postoperatively.

Following the surgical excision, the patient was instructed to adhere to a regimen of 5 weeks of complete activity restriction. At the 3‐week follow‐up, the incision was observed to be well healed, with no evidence of surrounding swelling, tenderness, or erythema. Notably, dorsiflexion showed significant early improvement. The patient continued to remain nonweight bearing for an additional 2 weeks before transitioning to a boot and weight bearing as tolerated. At the 8‐week follow‐up, the patient gradually progressed to full weight bearing over a timespa of 3 weeks. Physical examination at this stage demonstrated full range of motion, intact motor functions, and normal sensory nerve distributions, with the exception of cutaneous hyperesthesia at the surgical incision site. There were no signs of recurrence, malignant transformation, or other postoperative complications, confirming a favorable outcome 8‐weeks post‐op. At the 3‐month postoperative visit, the patient demonstrated complete resolution of symptoms, full ankle and subtalar range of motion, and a normal gait pattern. Given the excellent clinical recovery, the patient was released to follow‐up on an as‐needed basis. Subsequently, radiographs obtained by the patient′s primary care provider approximately 1.5 years postoperatively demonstrated no evidence of lesion recurrence or joint space compromise (Figure 8).

## 4. Discussion

Osteochondroma is the most common benign bone tumor; however, its presentation in the small bones of the appendicular skeleton, particularly the talus and subtalar joint, is quite rare. Although most cases of osteochondroma are asymptomatic, symptomatic presentations can lead to joint deformity, pain, swelling, tarsal tunnel syndrome, gait alterations, and a limited range of motion [3, 4, 6, 8, 9]. Surgical resection has proven to be an effective treatment for symptomatic osteochondromas [1, 4–7, 9].

Imaging, histology, and presentation play a critical role in diagnosing osteochondromas and differentiating these lesions from Trevor′s disease in pediatric patients. A comprehensive comparison between the two clinical pathologies can be found in Table 1. Important differential diagnoses to consider also include myositis ossificans, tumoral calcinosis, capsular chondroma, para‐articular chondroma, and synovial osteochondromatosis [14]. Isolated osteochondromas in the small joints of pediatric populations frequently present similarly to Trevor′s disease. Trevor′s disease, also known as dysplasia epiphysealis hemimelica, is a skeletal developmental disorder characterized by asymmetric dysplastic lesions of cartilage overgrowth in the epiphyses of long bones [11, 12]. The disease is caused by abnormal control of cell proliferation at affected epiphyses following endochondral ossification. Trevor′s disease is characterized by multiple epiphyseal exostosis throughout the entire body, with the most common exostosis being found in multiple joints of the lower limbs [13]. The current standard for the diagnosis of Trevor′s disease involves x‐ray and CT imaging, which demonstrate medullary and cortical continuity of the lesion with the adjacent epiphysis [14]. Notably, CT imaging demonstrating increased osteal growth from the epiphysis with a cartilaginous cap enhances diagnostic confidence [1, 10, 14]. On histology, thick, disorganized chondrocytes may be identified in the cap of the lesion; however, Trevor′s disease will lack any organization of these chondrocytes into structures resembling the growth plate [15]. In the present case, histopathologic examination of the excised lesion confirmed the diagnosis of osteochondroma. Routine histologic evaluation demonstrated features consistent with a benign cartilage‐capped osseous outgrowth, without evidence of malignancy or borderline cartilaginous transformation. Although immunohistochemical analysis for collagen Typ II and X expression may serve as an adjunctive diagnostic tool in select cases [15], it was not performed in this instance, as diagnostic confirmation was achieved through conventional histopathology.

**Table 1:** Comparison of solitary osteochondroma and Trevor′s disease including clinical presentation, pathology, and management.

Characteristics	Trevor′s Disease (dysplasia epiphysealis hemimelica)	Osteochondroma
Pathology	Asymmetric dysplastic lesions of cartilage overgrowth in epiphyses [11, 12].	Cartilage‐capped bony outgrowth from metaphysis [1, 2].
Prevalence	Rare, typically affects children < 10 years old [1, 2].	Most common benign bone tumor, affects < 20 years old [1, 2].
Common locations	Lower limb joints, particularly knees and ankles [13].	Long bones (distal femur and proximal tibia); rare in talus [1, 2].
Radiological features	Irregular lesion with medullary and cortical continuity with epiphysis; dysplastic cartilaginous cap [1, 10, 14].	Pedunculated or sessile outgrowth from metaphysis; osseous matrix with chondroid cap [1, 10, 14].
Histology and immunohistochemistry differentiation	Chondrocyte clusters with fibrillary matrix, thick disorderly cartilage cap, ossification centers with unabsorbed cartilage. Weak expression of Type II collagen, no expression of Collagen X [15].	Histological characteristics consistent with normal growth plate. Type II collagen expression in extracellular matrix. Strong expression of Collagen X in matrix [15].
Clinical presentation	Joint deformity, pain, limited range of motion, can cause limb length discrepancies and angular joint deformities [16, 17].	Often asymptomatic; symptomatic cases cause joint deformity, pain, stiffness, limited range of motion, and altered gait mechanics [3–7].
Treatment	Surgical resection for symptomatic lesions; conservative for mild cases. Biologic treatments to prevent heterotrophic ossification may be indicated in recurrent cases [18, 19].	Surgical excision for symptomatic lesions; conservative for asymptomatic [2].
Complications	Joint instability, recurrence postresection [12, 13].	Malignant transformation (< 1% cases), fracture, recurrence [6, 7, 10].
Prognosis	Good with surgical excision if joint integrity is preserved [13].	Excellent postresection; recurrence rare with complete excision [18].
Malignant transformation	No evidence of malignant transformation to chondrosarcoma yet reported [19, 20].	Extremely rare, but more likely transformation to secondary chondrosarcoma [21].

Histopathologic findings correlated with characteristic radiologic features, including cortical and medullary continuity with the parent bone, a well‐defined cartilage cap, metaphyseal origin, and absence of epiphyseal involvement [14, 15]. The concordance of clinical presentation, imaging findings, and histopathologic evaluation confirmed the diagnosis of osteochondroma.

For nonsymptomatic lesions, conservative interventions are recommended, including careful observation, pain management for symptomatic relief, and physical therapy to improve mobility and strength [22]. Additional treatment options for recurrent osteochondromas may include biologic agents to inhibit abnormal cartilage development and heterotrophic ossification, which may be more relevant in MHE syndromes or Trevor′s disease [18, 19, 23]. In this case, surgical excision was indicated due to significant alterations in joint range of motion, gait mechanics, and deformity. Surgical excision guidelines for isolated osteochondromas include complete resection to the bone base, with removal of the cartilage cap and perichondrium, ultimately lowering the risk of recurrence [2, 18]. In children, excision may be held until skeletal maturity to prevent early recurrence or damage to the physeal plate [18]. Surgical excision was elected in this case due to the symptomatic presentation of the lesion. In this patient, the lesion was loose in the sinus tarsi with well‐delineated margins, making complete surgical excision relatively simple. No additional bone was resected during the procedure to prevent possible destabilization of the joint. Previous cases have shown successful outcomes with surgical treatment of symptomatic osteochondromas of the talus [2, 18]. Potential complications of this procedure include recurrence of the osteochondroma, talar fracture, neurovascular injury, joint instability, stiffness, infection, bursa formation, subtalar degeneration, and incomplete resection [1, 18].

Recurrence following complete excision of a solitary osteochondroma is relatively uncommon, with reported rates generally less than 2%, and is most often attributed to incomplete removal of the lesion′s cartilage cap [18], [24]. Prolonged surveillance may be considered in skeletally immature patients, although due to the rarity of solitary osteochondroma of the subtalar joint in this age group, a standard duration of follow‐up has not yet been defined [8], [25]. In this case, the patient′s clinical improvement and absence of symptoms by 3 months supported discharge to follow‐up on an as‐needed basis. Clinical guidelines support short‐term follow up for pediatric osteochondroma in the absence of associated bony deformity or growth plate compromise [26].

## 5. Conclusion

Osteochondromas of the talus prove to be rare, specifically in patients who have not yet reached adolescence. These rare presentations emphasize the need for thorough diagnostic evaluation in any pediatric patient with a symptomatic foot mass. With complete symptom regression at 8 weeks following excision and lack of radiologic evidence of recurrence at 1.5 years postoperatively, this case underscores the importance of developing a reliable standard for accurate diagnosis, preoperative planning, and surgical treatment of symptomatic lesions. Early surgical intervention for symptomatic talar osteochondromas can restore function and prevent long‐term gait abnormalities. Additionally, thorough and multimodal preoperative imaging proved valuable in surgical planning by delineating lesion margins and aiding attempts at joint preservation throughout surgical excision. This case contributes to the limited literature on the diagnosis, management, and outcomes of osteochondromas of the talus in the pediatric population.

## Funding

No funding was received for this manuscript.

## Consent

This case report was deemed exempt from Institutional Review Board review as it does not meet the definition of human subjects research. All information has been deidentified in accordance with HIPAA and ProMedica Health Systems institutional policies. No identifiable patient information is presented, and therefore patient consent was not required in accordance with specific institutional policy.

## Conflicts of Interest

The authors declare no conflict of interest.

## Supporting information


**Supporting Information** Additional supporting information can be found online in the Supporting Information section. CARE criteria checklist.

## Data Availability

The data that support the findings of this study are available on request from the corresponding author. The data are not publicly available due to privacy or ethical restrictions.
