# Supplementary material for: Differentiation of Pediatric Osteochondroma From Trevor′s Disease and Successful Surgical Management: A Case Report
Source: Case Rep Orthop. 2026 Apr 18;2026:6640185. doi: 10.1155/cro/6640185 (PMC13090676; doi:10.1155/cro/6640185)
Supplement: Supplementary file 1 — Supporting Information Additional supporting information can be found online in the Supporting Information section. CARE criteria checklist. [file CRO-2026-6640185-s001.pdf]

CARE Checklist of information to include when writing a case report

CARE case report guidelines

(cc) BY-NC-ND

Topic	Item	Checklist item description	Reported on Line
**Title**	1	The diagnosis or intervention of primary focus followed by the words "case report"	4
**Key Words**	2	2 to 5 key words that identify diagnoses or interventions in this case report, including "case report"	32
**Abstract (no references)**	3a	Introduction: What is unique about this case and what does it add to the scientific literature?	34
	3b	Main symptoms and/or important clinical findings	54
	3c	The main diagnoses, therapeutic interventions, and outcomes	66
	3d	Conclusion—What is the main "take-away" lesson(s) from this case?	129
**Introduction**	4	One or two paragraphs summarizing why this case is unique (**may include references**)	88
**Patient Information**	5a	De-identified patient specific information.	53
	5b	Primary concerns and symptoms of the patient.	54
	5c	Medical, family, and psycho-social history including relevant genetic information	53
	5d	Relevant past interventions with outcomes	N/a
**Clinical Findings**	6	Describe significant physical examination (PE) and important clinical findings.	59
**Timeline**	7	Historical and current information from this episode of care organized as a timeline	159
**Diagnostic Assessment**	8a	Diagnostic testing (such as PE, laboratory testing, imaging, surveys).	61
	8b	Diagnostic challenges (such as access to testing, financial, or cultural)	N/a
	8c	Diagnosis (including other diagnoses considered)	106
	8d	Prognosis (such as staging in oncology) where applicable	46
**Therapeutic Intervention**	9a	Types of therapeutic intervention (such as pharmacologic, surgical, preventive, self-care)	66
	9b	Administration of therapeutic intervention (such as dosage, strength, duration)	67
	9c	Changes in therapeutic intervention (with rationale)	N/a
**Follow-up and Outcomes**	10a	Clinician and patient-assessed outcomes (if available)	72
	10b	Important follow-up diagnostic and other test results	73
	10c	Intervention adherence and tolerability (How was this assessed?)	71
	10d	Adverse and unanticipated events	78
**Discussion**	11a	A scientific discussion of the strengths AND limitations associated with this case report	121
	11b	Discussion of the relevant medical literature **with references**.	81
	11c	The scientific rationale for any conclusions (including assessment of possible causes)	133
	11d	The primary "take-away" lessons of this case report (without references) in a one paragraph conclusion	129
**Patient Perspective**	12	The patient should share their perspective in one to two paragraphs on the treatment(s) they received	N/a
**Informed Consent**	13	Did the patient give informed consent? Please provide if requested	Yes ☐ No ☐ IRB exempt
